# Host biomarkers distinguish dengue from leptospirosis in Colombia: a case–control study

**DOI:** 10.1186/1471-2334-14-35

**Published:** 2014-01-20

**Authors:** Andrea L Conroy, Margarita Gélvez, Michael Hawkes, Nimerta Rajwans, W Conrad Liles, Luis Angel Villar-Centeno, Kevin C Kain

**Affiliations:** 1 Sandra A. Rotman Laboratories, Sandra Rotman Centre, University Health Network-Toronto General Hospital, University of Toronto, Toronto M5G 1 L7, Canada; 2 Department of Laboratory Medicine and Pathobiology, University of Toronto, Toronto M5S 1A8, Canada; 3 Centro de Investigaciones Epidemiológicas, Facultad de Salud, Universidad Industrial de Santander, Bucaramanga Dept 680002, Colombia; 4 Tropical Disease Unit, Division of Infectious Diseases, Department of Medicine, University of Toronto, Toronto M5G 2C4, Canada; 5 Department of Medicine, University of Washington, Seattle, WA 98195, USA; 6 Sandra Rotman Centre, Suite 10–351, Toronto Medical Discovery Tower, MaRS Centre, 101 College St., M5G1L7 Toronto, Canada

**Keywords:** Dengue fever, Leptospirosis, Acute febrile illness, Host biomarkers, Clinical discrimination, Combinatorial models

## Abstract

**Background:**

Dengue fever and leptospirosis have partially overlapping geographic distributions, similar clinical presentations and potentially life-threatening complications but require different treatments. Distinguishing between these cosmopolitan emerging pathogens represents a diagnostic dilemma of global importance. We hypothesized that perturbations in host biomarkers can differentiate between individuals with dengue fever and leptospirosis during the acute phase of illness.

**Methods:**

We randomly selected subjects from a prospective cohort study of acute febrile illness in Bucaramanga, Colombia and tested 19 serum biomarkers by ELISA in dengue fever (DF, n = 113) compared to subjects with leptospirosis (n = 47). Biomarkers were selected for further analysis if they had good discriminatory ability (area under the ROC curve (AUC) >0.80) and were beyond a reference range (assessed using local healthy controls).

**Results:**

Nine biomarkers differed significantly between dengue fever and leptospirosis, with higher levels of Angptl3, IL-18BP, IP-10/CXCL10, Platelet Factor 4, sICAM-1, Factor D, sEng and sKDR in dengue and higher levels of sTie-2 in leptospirosis (p < 0.001 for all comparisons). Two biomarkers, sEng and IL18BP, showed excellent discriminatory ability (AUROC >0.90). When incorporated into multivariable models, sEng and IL18BP improved the diagnostic accuracy of clinical information alone.

**Conclusions:**

These results suggest that host biomarkers may have utility in differentiating between dengue and leptospirosis, clinically similar conditions of different etiology.

## Background

Dengue virus infection and leptospirosis represent important causes of acute febrile illness whose diagnosis and management in resource-poor settings remains challenging. Both diseases are potentially fatal, and represent important causes of morbidity and mortality globally. As emerging or re-emerging vector- and water-borne pathogens, respectively, dengue and leptospirosis are increasingly important considerations in patients with acute febrile illness, particularly in the context of decreasing malaria transmission in many areas of the world [1].

The burden of illness attributable to dengue viruses is estimated to be 390 million annually [2]. An estimated 2.5-4 billion people are at risk of infection, and transmission has increased in recent years, such that dengue is re-emerging as an international public health concern [3]. Dengue viruses are members of the flavivirus family and consist of four distinct serotypes. Transmission occurs in tropical and sub-tropical regions around the world through the bite of the *Aedes* mosquito vector. Clinical manifestations range from: (1) asymptomatic infection; to (2) a non-specific febrile illness (dengue fever); to (3) a life-threatening complication, severe dengue, often characterized by plasma leakage and coagulopathy. The pathogenesis of severe dengue is the result of a complex interaction of viral and host factors. The risk of severe dengue is increased 40–80 fold after a second infection with a different serotype [4,5], which may be explained by non-neutralizing heterotypic antibody-mediated enhancement of infection. Complement activation by virus-antibody complexes and T-cell mediated immunopathology have also been implicated in the progression of secondary dengue infection. Laboratory confirmation of infection relies on serologic methods, viral culture, and/or PCR and is not generally rapidly available to assist clinical decision-making, particularly in endemic resource-constrained areas. Specific anti-viral therapy is not available, but intensive supportive care may reduce mortality in severe dengue from 20% to less than 1% [6].

Leptospirosis accounts for a significant proportion of febrile illness among hospitalized patients in Asia and the Americas [7-9]. Pathogenic spirochetes of the genera *Leptospira* infect humans through contact of skin or mucous membranes with water or soil contaminated with the urine of host animals, who harbor the bacteria within their renal tubules. Outbreaks have been reported after monsoons and natural disasters [10], as well as among adventure travelers with prolonged water contact [11]. As in dengue, clinical manifestations vary widely across a spectrum of disease severity. The majority of infections produce asymptomatic seroconversion or undifferentiated fever; however, severe disease may develop in 5-10% of symptomatic patients, evidenced by renal and pulmonary involvement (Weil’s disease). The case-fatality rate may be as high as 5-15% when multisystem complications occur [12]. Serologic methods are used in clinical settings for a microbiologic diagnosis of leptospirosis, meaning that timely confirmation of infection is not widely available. The treatment involves specific antibiotic therapy, such that early diagnosis can assist treatment decisions and may arrest disease progression. Leptospirosis is often misdiagnosed as dengue fever, and is frequently under-recognized in endemic regions and during outbreaks [8,13].

Host immunopathology plays an important role in the progression of both dengue and leptospirosis [14,15]. Indeed, severe infection across a range of viral, bacteria and parasitic pathogens is characterized by engagement of shared host defense pathways, including inflammation, angiogenesis, coagulation and endothelial activation [16-18]. We hypothesized that peripheral blood biomarkers of these pathways may have clinical utility as diagnostic tools to differentiate between dengue and leptospirosis. We applied combinatorial approaches [17], using information provided by clinical features, standard clinical laboratory tests and multiple biomarkers from distinct pathobiological pathways, to examine novel strategies to distinguish dengue from leptospirosis.

## Methods

### Ethics statement

Ethical approval for this study was granted from the Medical Ethics Committee of the Universidad Industrial de Santander in Bucaramanga, Colombia. All subjects (or parents/guardians for minors) provided written informed consent to enter the study.

### Study design

This study was a case–control study nested within a prospective cohort study of subjects with suspected dengue fever in Bucaramanga, Colombia. Cases and controls were randomly chosen using simple randomization from the cohort database following identification of subjects with available serum samples that had not been previously freeze-thawed. Cases were defined as individuals with dengue infection and controls were individuals with leptospirosis.

### Study population

Participants greater than five years of age with an acute febrile syndrome less than 96 hours were enrolled in a prospective outpatient cohort study examining predictors of disease severity in dengue fever. Following enrollment, a physical examination was performed and a blood sample was collected to determine levels of hematocrit and albumin and to assess platelet and leukocyte counts. An additional serum sample was collected and stored at -80C for future biomarker assessment. Subjects were excluded based on the presence of the following conditions: history of concomitant diseases such as diabetes, acquired immunodeficiency syndrome, hematologic disorders, cancer, or cardiac disease; dengue hemorrhagic fever at baseline, major bleeding, hypoalbuminemia (<3 g/dL), effusions, or shock.

### Study definitions

#### ***Dengue fever***

Diagnosis of dengue infection was made based on either: viral isolation, a shift from a negative to a positive IgM test result, or a four-fold increase in existing dengue antibodies from admission to convalescence (7–15 days following symptom onset). Study subjects with negative convalescent IgM test were considered to have another cause for fever. We were not able to differentiate between primary and secondary dengue infections in this study.

#### ***Leptospirosis***

In subjects negative for dengue fever a diagnosis of leptospirosis was made based on a shift from negative to positive IgM test result in admission and convalescence samples, or a four-fold increase in leptospirosis antibody titers.

#### ***Healthy controls***

Serum samples were collected from 15 healthy adults from Bucaramanga to derive a population-based normal range. Samples were not tested for past dengue virus or leptospirosis infection, but all controls were asymptomatic at the time of blood sampling and were unlikely to have an acute infection.

### Biomarker assessment

Nineteen biomarkers were selected because they represent markers of novel pathways implicated in infectious disease pathobiology, including inflammation, coagulation, and endothelial activation [19-26]. These proteins provide information about a broad range of innate immune responses and serve to characterize the host response to different pathogens. Serum concentrations of biomarkers were measured using ELISA DuoSets from R&D Systems (Minneapolis, MN). Biomarkers measured were: angiopoietin (Ang)-1, Ang-2, soluble Tie-1 (sTie-1), soluble Tie-2 (sTie-2), vascular endothelial growth factor (VEGF), soluble VEGFR-1/Flt-1(sFlt-1), soluble VEGR-2/KDR (sKDR), soluble Endoglin (sEng), C-reactive protein (CRP), interleukin-10 (IL-10), interleukin-18 binding protein (IL-18BP), 10 kDa-interferon induced protein (IP-10, CXCL10), soluble ICAM-1, chitinase 3-like 1 (CHI3L1), complement factor 5a (C5a), complement factor D (Factor D), angiopoietin-like 3 (Angptl3), and angiopoietin-like 4 (Angptl4). All ELISA kits were validated prior to use and appropriate samples dilutions were obtained for each biomarker by testing a dilution curve of serum obtained from febrile subjects. ELISAs were performed as previously described [16] and biomarker concentrations were extrapolated using a 4-parameter logistic slope curve (GraphPad Prism v5.0).

### Statistical analysis

GraphPad Prism v5, SPSS v16 and MedCalc software were used for statistical analysis. Comparisons of continuous variables were performed using the Mann–Whitney *U* test. Bonferonni adjustment was used to account for multiple testing. Receiver operating characteristic (ROC) curves were used to assess the discriminatory ability of the biomarkers. The area under the ROC curves (AUC) or c-indices (ROC generated from the predicted probabilities of logistic regression models) were compared using the Delong-Delong Clarke Pearson method [27]. Biomarker cut-points were established by using the Youden index.

Comparisons of proportions were performed using Pearson chi-square test or Fisher’s exact test, as appropriate. Adjusted odds ratios were calculated using logistic regression. Variables were selected for inclusion in logistic regression models using forward stepwise logistic regression. All logistic regression models were validated by ensuring the Hosmer-Lemeshow goodness-of-fit test was not significant (p > 0.05). Complete model validation is provided as an Additional file 1: Table S1 and Table S2.

## Results

### Study population

160 subjects between the ages of 5 and 81 years with an acute febrile illness were included in the study. The median age for individuals with dengue fever and leptospirosis was 25 and 27 years respectively. There was an equal distribution of dengue and leptospirosis in both men and women. The demographic and clinical characteristics and laboratory findings at presentation are summarized in Table 1.

**Table 1:** Demographic and clinical characteristics of population

	**Dengue (n = 113)**	**Leptospirosis (n = 47)**	**P value**
**Demographic characteristics**			
Age, years	25.0 (16.0–41.0)	27.0 (20.0–38.0)	0.370
Sex, number (% M)	52 (46.0)	26 (55.3)	0.284
Height, cm	163 (152–170)	164 (157–174)	0.050
Weight, kg	61.0 (50.5–74.5)	62.0 (51.5–71.0)	0.821
Body mass index (kg/m^2^)	23.2 (19.7–26.9)	21.7 (19.6–24.5)	0.213
Duration of fever, hours	77.0 (64.0–88.8)	70.5 (55.0–79.7)	0.042
**Laboratory findings**			
Axillary temperature,ºC	36.5 (36.0–37.4)	36.0 (35.6–36.5)	0.003
Number (%) >38ºC	12 (10.6)	3 (6.5)	0.557
Platelet count (x10^3^)	126 (83–180)	202 (175–240)	<0.001
Number (%) <100 x10^3^/uL	33 (30)	2 (5.7)	<0.001
Leukocyte count	2900 (2200–3950)	4900 (3600–6300)	<0.001
Number (%) <5000/uL	99 (90.0)	21 (48.8)	<0.001
Hematocrit	38.7 (35.9–42.2)	41.1 (36.7–44.7)	0.062
Positive tourniquet test	75 (66.4)	23 (48.9)	0.039
**Signs and symptoms, number (%)**			
Headache	107 (94.7)	45 (95.7)	0.780
Retro-orbital pain	79 (69.9)	31 (66.0)	0.623
Asthenia	78 (69.0)	37 (78.7)	0.214
Muscle pain	102 (91.1)	43 (91.5)	0.932
Joint pain	90 (79.6)	35 (74.5)	0.576
Chills	107 (94.7)	44 (93.6)	0.788
Cough	41 (37.6)	22 (46.8)	0.283
Nasal congestion	38 (34.9)	22 (46.8)	0.159
Sore throat	36 (31.9)	26 (55.3)	0.006
Rash	48 (42.5)	7 (14.9)	<0.001
Facial erythema	57 (50.4)	7 (14.9)	<0.001
Pruritis	35 (32.1)	11 (23.4)	0.274
Nausea	86 (76.1)	37 (78.7)	0.721
Vomiting	38 (33.6)	17 (36.2)	0.758
Diarrhea	42 (37.2)	11 (23.4)	0.092
Abdominal pain	61 (54.5)	25 (53.2)	0.883
Blurred Vision	46 (41.1)	17 (36.2)	0.564
Dizziness	75 (67.0)	40 (85.1)	0.020
Drowsiness	68 (60.7)	28 (59.6)	0.893
Dehydrated	66 (60.6)	32 (69.6)	0.288
Conjunctival injection	48 (42.5)	11 (23.4)	0.023
Orthostatic hypotension	17 (15.0)	2 (4.3)	0.055
Hepatomegaly	1 (0.9)	5 (10.6)	0.003

Data are presented as median (interquartile range) unless otherwise indicated.

Group comparison by Chi-square or Fisher’s exact test (where <5 cases in any specified cell) for dichotomous variables or Mann–Whitney for continuous variables.

### Clinical and laboratory factors that discriminate between dengue fever and leptospirosis

To identify clinical signs and laboratory parameters that could aid in differentiating dengue fever from leptospirosis, we generated adjusted odds ratios for variables with p < 0.10 by bivariate analysis. Following adjustment for age, sex, height, and duration of illness, thrombocytopenia and leukopenia were independently associated as risk factors for dengue fever with adjusted odds ratios (aOR) of 10.0 (95% CI, 2.2-45.8), p = 0.003 and 9.3 (95% CI, 3.6-24.0), p < 0.001 respectively. In addition, the presence of rash (aOR (95% CI), p-value: 5.8 (2.1-15.7), p = 0.001) facial erythema (7.3 (2.8-19.3), p < 0.001) and conjunctival injection (3.0 (1.3-7.1), p = 0.012) were more common in subjects with dengue fever. Dizziness (aOR (95% CI), p-value: 0.4 (0.2-1.0), p = 0.047) and sore throat (0.3 (0.1-0.7), p = 0.004) were more common in leptospirosis compared to dengue fever.

**Figure 1:**
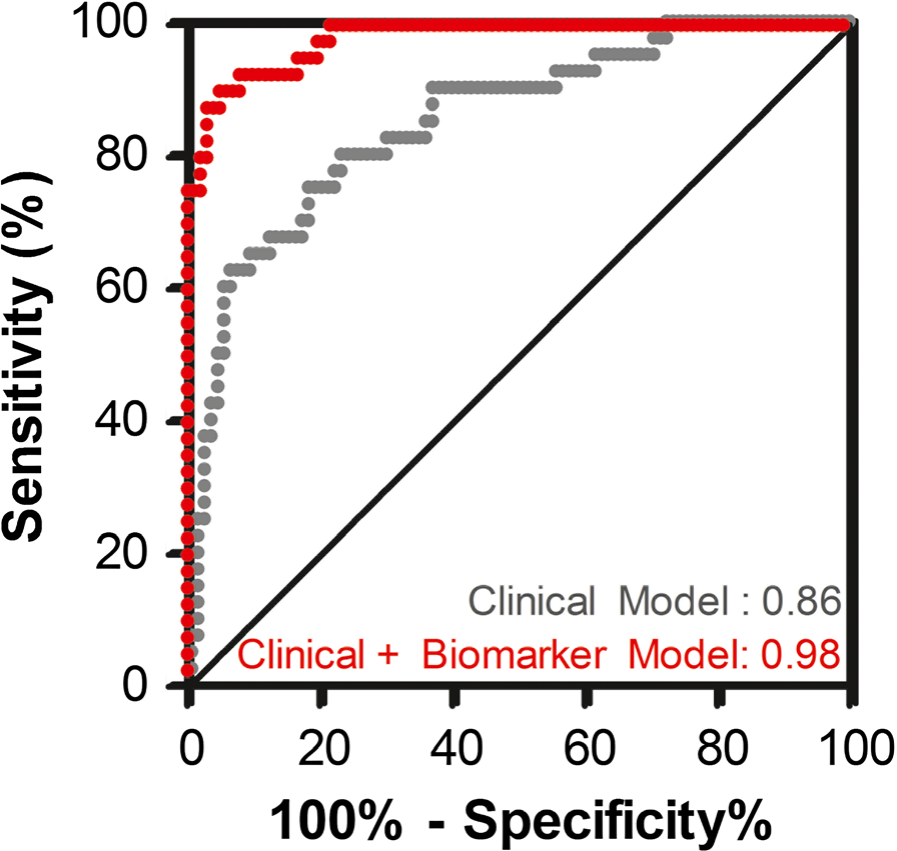
**Integrating Clinical and Laboratory Data with Biomarker Data Improves Discrimination of Dengue Fever and Leptospirosis.** Logistic regression analysis was used to generate two models to discriminate between dengue fever and leptospirosis and the predicted probabilities from those models were plotted using ROC curve analysis. The first model used clinical and laboratory data (clinical model: age, sex, height, duration of illness, leukopenia, rash, dizziness) and had good discriminatory performance with a c-index (equivalent to the AUC) of 0.86 (95% CI: 0.79-0.91). By adding in the biomarker data, we generated a model with excellent discriminatory ability and a c-index of 0.979 (95% CI: 0.94-0.996). The biomarker model (clinical model with IL-18BP, sEng) had a c-index that was statistically higher than that of the clinical model (p = 0.0003).

To further explore how clinical and laboratory parameters could be incorporated into a model to differentiate between dengue fever and leptospirosis, we used logistic regression and forward step-wise selection to identify variables with the best discriminatory ability. All factors with significant adjusted odds ratios from Table 2 were included into logistic regression models and leukopenia, rash and dizziness were identified as the best three discriminatory measures. A clinical model including age, sex, height, duration of illness, leukopenia, rash and dizziness had a c-index (equivalent to the AUC) of 0.86 indicating that these parameters have good discriminatory ability (Figure 1, Additional file 1: Table S1).

**Table 2:** Clinical and laboratory factors associated with dengue fever, relative to leptospirosis

	**Odds Ratio (95% CI)**	**P-Value**	Adjusted OR (95% CI)*	P-value*
Positive tourniquet test	1.4 (0.9–2.3)	0.156	1.4 (0.7–2.9)	0.354
Thrombocytopenia	6.2 (1.6–24.3)	0.001	10.0 (2.2–45.8)	0.003
Leukopenia	3.8 (2.4–6.0)	<0.001	9.4 (3.7–23.8)	<0.001
Orthostatic hypotension	3.0 (0.8–11.5)	0.055	3.4 (0.7–15.9)	0.117
Rash	3.1 (1.5–6.5)	<0.001	5.8 (2.1–15.7)	0.001
Facial erythema	3.8 (1.8–8.0)	<0.001	7.3 (2.8–19.3)	<0.001
Conjunctival injection	1.9 (1.1–3.4)	0.023	3.0 (1.3–7.1)	0.012
Diarrhea	1.6 (0.9–2.9)	0.092	1.9 (0.8–4.4)	0.125
Dizziness	0.5 (0.2–0.9)	0.020	0.4 (0.2–1.0)	0.047
Hepatomegaly	0.3 (0.2–0.5)	0.003	0.1 (0.01–1.4)	0.093
Sore throat	0.5 (0.3–0.8)	0.006	0.3 (0.1–0.7)	0.004

*Adjustment for: age, sex, height, duration of illness (logistic regression analysis). Model fit was assessed using the Hosmer-lemeshow test and ensuring the term was insignificant (p > 0.05).

**Figure 2:**
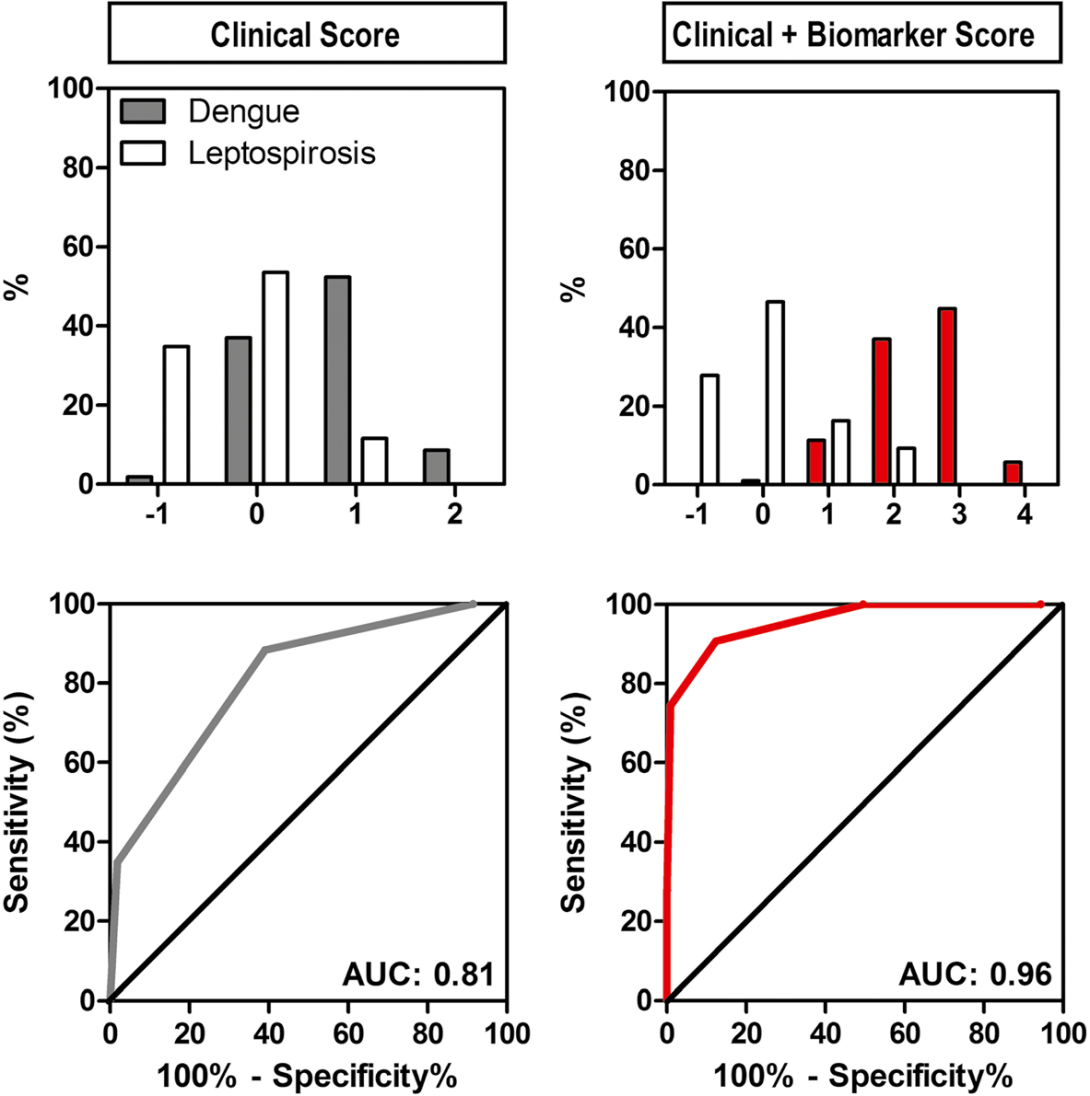
**Integrating Biomarker Data into a Clinical Score Improves Diagnosis of Dengue Fever.** A clinical score (from -1 to 2) was created for each study participant by assigning a value of 0 (not present), -1 (more common in leptospirosis) or +1 (more common in dengue) for leukopenia, rash and dizziness. The score was used to create an area under the ROC curve (AUC) of 0.81 (95% CI, 0.73-0.86), p < 0.001. Biomarker data were then integrated into the clinical score (from -1 to 4) by assigning a value of +1 if IL-18BP (>24.5 ng/mL) and sEng (> 9.12 ng/mL) levels were higher than the assigned cut-offs to generate an AUC of 0.96 (95% CI, 0.91-0.98), p < 0.001.

Taking another more intuitive approach we used the clinical parameters identified by logistic regression to generate a clinical score that could easily be implemented and interpreted to identify subjects with dengue fever. For each dichotomous measure, we assigned a value of 0 (not present), -1 (more common in leptospirosis) or +1 (more common in dengue fever) for each variable (Figure 2). Thus, using a clinical score ranging from -1 to 2, leukopenia, rash and dizziness were able to differentiate between dengue fever and leptospirosis with an AUC (95% CI) of 0.81(0.73-0.87), p < 0.001. Using a cut-point of ≥1, this model had a sensitivity of 61%, a specificity of 88% and a positive and negative likelihood ratio of 5.2 and 0.4 respectively to correctly diagnose dengue fever.

### Host biomarkers differentiate between dengue fever and leptospirosis

We hypothesized that host biomarkers derived from pathways of disease pathogenesis in dengue may further improve discrimination between these clinically non-specific acute febrile syndromes. We examined 19 different serum biomarkers from different pathways implicated in dengue pathogenesis focusing on: endothelial activation and angiogenesis (Ang-1, Ang-2, sTie-2, sTie-1, VEGF, sFlt-1, sFlk-2, sEng, angiopoietin-like 4), inflammation (CRP, IL-10, IL-18BP, IP-10/CXCL10, sICAM-1, CHI3L1), complement activation and coagulation (C5a, Factor D, PF4) and the regulation of lipids (angiopoietin-like 3). Data are summarized in Table 3.

**Table 3:** Biomarkers levels measured at time of presentation in dengue fever and leptospirosis

	**Healthy control (n = 15)**	**Dengue (n = 113)**	**Leptospirosis (n = 47)**	**P value**
				**Dengue vs. Leptospirosis**
Angiopoietin-1	44.9 (32.9-53.6)	32.3 (24.1-45.0)	35.8 (29.0-46.7)	0.236
Angiopoietin-2	1.4 (1.0-2.6)	1.8 (1.3-2.5)	2.0 (1.3-3.0)	0.474
sTie-1	8.3 (6.8-12.7)	10.1 (8.1-13.5)	9.0 (7.6-11.6)	0.120
VEGF	0.21 (0.15-0.28)	0.13 (0.05-0.30)	0.24 (0.08-0.49)	0.017
sFlt-1	0.04 (0.04-0.34)	0.04 (0.04-0.88)	0.15 (0.04-0.70)	0.864
C5a	18.5 (13.8-22.1)	53.8 (34.3-63.8)	41.2 (30.2-59.6)	0.103
CRP ^a^	1.8 (1.2-6.1)	19.7 (9.1-41.4)	13.3 (4.1-39.8)	0.125
IL-10	0.02 (0.02-0.16)	0.09 (0.02-0.37)	0.08 (0.02-0.20)	0.688
CHI3L1	44.5 (35.8-61.3)	58.1 (43.0-78.6)	52.1 (36.5-74.1)	0.114
Angiopoietin-like 4	44.7 (30.2-90.9)	40.4 (30.6-57.1)	49.8 (35.9-67.8)	0.094
Angiopoietin-like 3	108 (83–118)	165 (141–195)	122 (96–148)	<0.001*
IL-18BP	5.8 (4.4-8.3)	67.8 (41.5-90.9)	11.4 (7.4 (21.5)	<0.001*
CXCL10	0.2 (0.2-0.4)	3.2 (1.2-4.4)	0.5 (0.2-1.4)	<0.001*
Platelet Factor 4 ^a^	18.5 (15.6-27.1)	25.2 (16.2-39.1)	19.1 (14.0-24.6)	<0.001*
sICAM-1	169 (136–187)	352 (300–488)	228 (159–275)	<0.001*
Factor D	1143 (1018–1265)	1376 (1172–1615)	970 (837–1128)	<0.001*
sEng	6.6 (5.7-8.4)	11.7 (9.6-13.9)	7.6 (7.2-8.2)	<0.001*
sKDR	5.3 (4.5-6.1)	6.1 (5.5-7.1)	5.2 (4.6-6.1)	<0.001*
sTie-2	8.7 (6.4-9.7)	7.7 (5.9-9.3)	10.0 (7.8-13.0)	<0.001*

Biomarker concentrations are presented as median (IQR) in ng/mL unless otherwise indicated, ^a^μg/mL.

*p < 0.05 following adjustment for 19 pair-wise comparisons.

**Figure 3:**
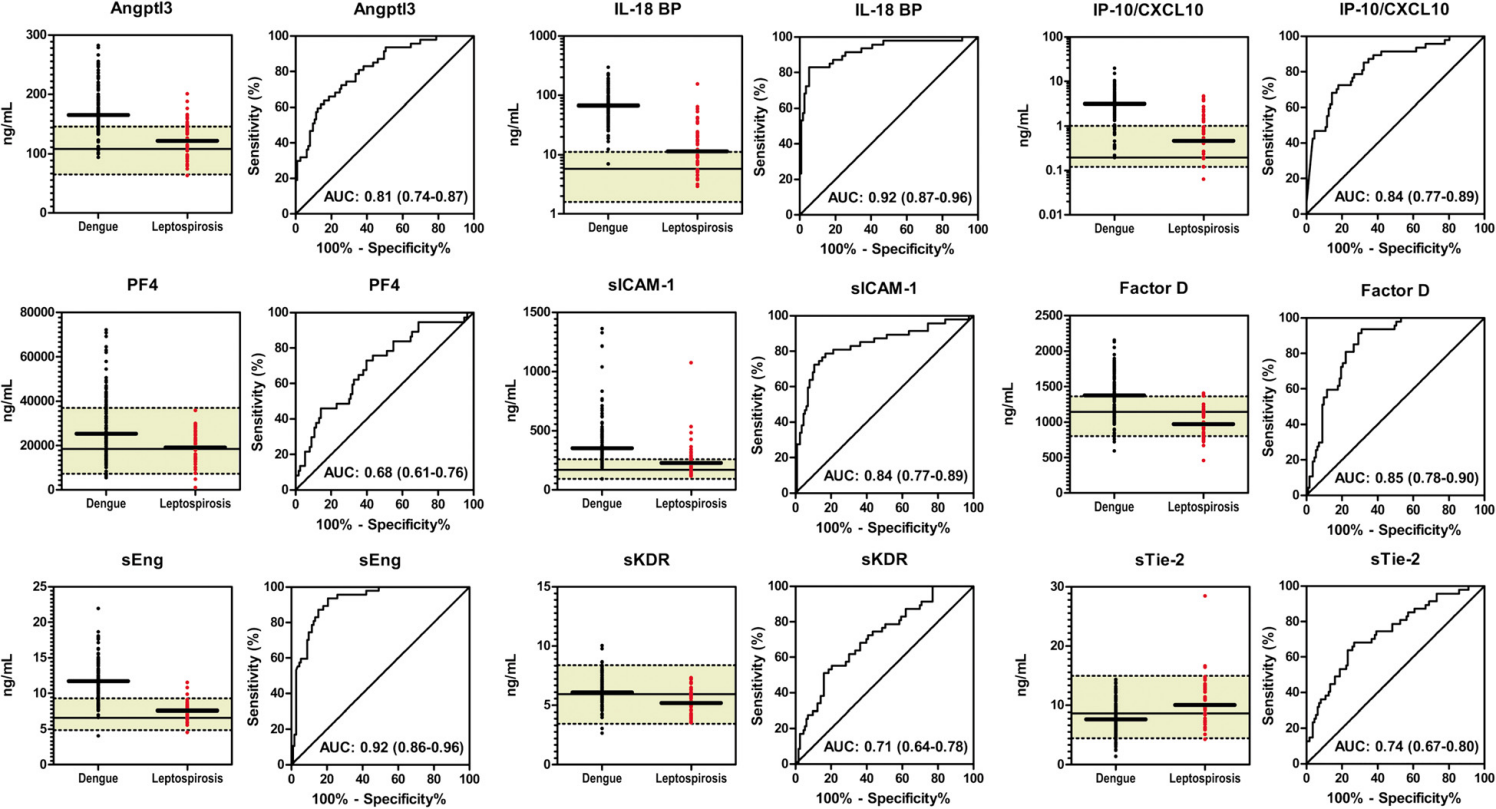
**Biomarkers Discriminate Between Dengue Fever and Leptospirosis.** Aligned dot plots and median of serum biomarker levels in dengue fever (n = 113) and leptospirosis (n = 47) measured at time of presentation during the acute phase of febrile illness. A population derived healthy range for adults in Bucaramanga, Colombia (n = 15) is represented by the shaded area with the median and 5-95% shown by the horizontal limits. All biomarkers were significantly different between cases with dengue fever and controls with leptospirosis (p < 0.001) following Bonferonni adjustment for multiple comparisons (19 pair-wise comparisons).

Of the 19 biomarkers measured, 9 biomarkers differed significantly between dengue fever and leptospirosis following Bonferonni correction for multiple testing. Median levels of Angptl3, IL-18BP, CXCL10, Platelet Factor 4, sICAM-1, Factor D, sEng and sKDR were higher in dengue fever compared to leptospirosis (p < 0.001 for all). sTie-2 was the only biomarker with median levels higher in subjects with leptospirosis compared to dengue fever. Data for these markers are shown in Figure 3, superimposed on population-derived normal range (median, 5-9%). Receiver operating characteristic (ROC) curves were generated to assess the discriminatory ability of the biomarkers (Figure 3) and the performance characteristics of the ROC curves are reviewed in Table 4.

**Table 4:** Performance characteristics of biomarkers significantly different in dengue fever following adjustment for multiple comparisons

	**AUC (95% CI)**	Cut-off*	**Sensitivity (95% CI)**	**Specificity (95% CI)**	**Positive LR (95% CI)**	**Negative LR (95% CI)**
Angiopoietin-like 3	0.81 (0.74–0.87)	>135.75	84.1 (76.0–90.3)	63.8 (48.5–77.3)	2.3 (1.8–2.9)	0.3 (0.1–0.5)
IL-18BP	0.92 (0.87–0.96)	>24.52	94.7 (88.8–98.0)	83.0 (69.2–92.4)	5.6 (4.9–6.4)	0.06 (0.02–0.2)
CXCL10	0.84 (0.77–0.89)	>0.96	82.3 (74.0–88.8)	72.3 (57.4–84.4)	2.9 (2.4–3.6)	0.2 (0.1–0.5)
Platelet Factor 4 ^a^	0.69 (0.61–0.76)	>29.98 ^a^	39.8 (30.7–49.5)	97.9 (88.7–99.9)	18.7 (14.9–23.6)	0.6 (0.1–4.3)
sICAM-1	0.84 (0.77–0.89)	>285.9	83.2 (75.0–89.6)	78.7 (64.3–89.3)	3.9 (3.3–4.6)	0.2 (0.1–0.4)
Factor D	0.85 (0.79–0.90)	>1248.1	69.0 (59.6–77.4)	93.6 (82.5–98.7)	10.8 (9.4–12.5)	0.3 (0.1–1.1)
sEng	0.92 (0.86–0.96)	>9.12	79.7 (71.0–86.6)	93.6 (82.5–98.7)	12.5 (11.1–14.1)	0.2 (0.07–0.7)
sKDR	0.71 (0.64–0.78)	>5.18	84.1 (76.0–90.3)	51.1 (36.1–65.9)	1.7 (1.3–2.3)	0.3 (0.2–0.5)
sTie-2	0.73 (0.65–0.80)	≤ 9.18	72.6 (63.4–80.5)	68.1 (52.9–80.9)	2.3 (1.8–2.9)	0.4 (0.2–0.7)

*Concentration in ng/mL unless otherwise indicated, ^a^μg/mL.

ROC curve analysis- non-parametric analysis by method of Delong et al. with the confidence interval estimated using binomial.

Although several biomarkers were able to discriminate between dengue fever and leptospirosis, the median biomarker levels in dengue fever or leptospirosis did not extend beyond the population derived normal range, suggesting that these biomarkers may not have clinical utility. There were five biomarkers where the median levels were outside the population-derived normal range in dengue fever, but not leptospirosis: Angptl3, IL-18BP, CXCL10, sICAM-1, and sEng. Two biomarkers had excellent discriminatory ability (AUC >0.90) and using the Youden index to dichotomize the biomarkers one had high sensitivity, IL-18BP (sensitivity: 94.7%) while the other had high specificity, sEng (specificity: 93.6%).

### Combining clinical and biomarker data improve discrimination

Since we had identified biomarkers with excellent discriminatory ability on their own, we wanted to explore whether these biomarkers could be integrated into the clinical models to improve clinical prediction. Intuitively, we assumed the two biomarkers with the best individual performance would be the best candidates to integrate into the clinical models. To test this assumption we used forward stepwise logistic regression and confirmed that IL-18BP and sEng were the best discriminatory biomarkers.

By adding the biomarkers into the clinical logistic regression model (clinical + biomarker model), we achieved a c-index (95% CI) of 0.98 (0.94-1.0), which was significantly better than the clinical model alone (p = 0.0003 comparing the AUCs by the method of Delong et al.) (Figure 1). Next, we added the biomarkers into the clinical score to generate a clinical + biomarker score that ranged from -1 to 4 (Figure 2). The AUC for the clinical + biomarker model was 0.96 (95% CI, 0.91-0.98) and at a cut-point ≥2 the model had a sensitivity of 87.6% (95% CI, 79.8-93.2) and specificity of 90.7 (77.9-97.4). This model had an AUC that was significantly higher than the AUC of the clinical model alone (0.96 vs. 0.81, p < 0.0001).

## Discussion

In areas where both dengue and leptospirosis co-circulate, misdiagnosis of these febrile illnesses is common, given the considerable overlap in clinical signs and symptoms [8,9,13,28]. The lack of affordable, timely and practical diagnostic tests for both dengue and leptospirosis in many settings contributes to the diagnostic dilemma. Previous studies have examined strategies to distinguish between dengue and leptospirosis on the basis of clinical and traditional laboratory parameters, including petechial counts on a standardized tourniquet test, total leukocyte count, aspartate transaminase, and albumin level [9,13]. Our study adds to this limited diagnostic toolbox, describing a panel of novel, clinically informative biomarkers to distinguish dengue from leptospirosis. Activation of specific host defense pathways appears to differ between the two infections, as indicated by differences in circulating concentrations of key regulatory proteins. Of note, this observation may be leveraged to generate clinically useful diagnostic tests based on host proteins that could complement or replace pathogen-based microbiologic diagnosis. To this end, we have taken preliminary measures to assess the clinical utility of these host biomarkers, demonstrating that, alone and in combination, biomarkers improve the ability to discriminate between dengue and leptospirosis above and beyond clinical information alone. Elevated levels of sEng and IL18BP point strongly to a diagnosis of dengue virus infection, rather than leptospirosis. Additional studies will be required to validate these findings in independent populations.

The primary objective of this work was to identify clinical and classical laboratory features, as well as novel biomarkers that can be used by clinicians faced with the diagnostic dilemma of distinguishing between dengue and leptospirosis. Thrombocytopenia, leukopenia, rash, facial erythema, conjunctival injection and a positive tourniquet test were more common among patients with dengue whereas dizziness and sore throat were reported more commonly by patients with leptospirosis. Similarly, previous comparative studies have documented lower total leukocyte counts and higher petechial counts on tourniquet test in dengue infection relative to leptospirosis [9,13]. Nonetheless, these features are variably present in both infections, such that clinical criteria alone, even when formalized into multivariate models and clinical scores, remained suboptimal for distinguishing between dengue and leptospirosis in our prospective sample. Information derived from circulating levels of novel biomarkers improved diagnostic accuracy, with sEng and IL18BP showing the best performance individually and in multivariable models to distinguish between dengue and leptospirosis. Furthermore, both of these biomarkers were markedly elevated relative to normal controls, suggesting they could be integrated into clinical practice as a diagnostic tool for dengue virus infection.

Host biomarkers sEng and IL-18BP compare favourably with another recently commercialized point-of-care diagnostic modality for dengue based on detection of the virus NS1 antigen. In our study, the sensitivity and specificity of elevated sEng for the diagnosis of dengue (relative to patients with leptospirosis) were 80% and 94%, and for IL-18BP were 95% and 83%, respectively, using optimal thresholds for the study population. In comparison, sensitivity of the NS1 antigen was lower (62% [29] and 69% [30] in previous reports). The specificity, however, of direct viral antigen detection was marginally higher (100% [29] and 96% [30] in these reports). Of note, our approach using host biomarker signatures for diagnosis contrasts with the more conventional approach of direct viral antigen detection.

Endoglin, a component of the tumor necrosis factor-beta (TGF-β) receptor complex, participates in angiogenic and inflammatory signaling pathways. Endoglin is abundantly expressed on endothelial cells and is upregulated during inflammation [31]. The soluble form of the receptor (sEng) is shed from the endothelial surface into the circulation in the setting of critical illness by cleavage through matrix metalloproteinases [31]. By indirect inhibition of TGF-β signaling, sEng has antiangiogenic effects, produces endothelial dysfunction with vascular leak, and abrogates anti-inflammatory effects of TGF-β1 [32,33]. Elevated levels of sEng have been found in patients with pre-eclampsia [32], as well as severe and placental malaria [34,35]. We examined sEng in patients with dengue or leptospirosis and found significantly elevated serum levels in the majority of patients with dengue. Upregulation and shedding of sEng into the peripheral circulation due to endothelial activation during dengue virus infection may account for this observation, and may be a relatively specific indicator of dengue infection, since levels were not elevated in leptospirosis compared to healthy controls. This finding suggests that sEng may have clinical utility as a diagnostic biomarker of dengue in populations where dengue and leptospirosis co-circulate. Mechanistically, high sEng levels may attenuate TGF-β1 mediated anti-inflammatory responses, which may contribute to the disease manifestations of severe dengue. Further studies are warranted to investigate a putative pathogenic role of sEng in dengue virus infection.

IL-18 is a proinflammatory cytokine that stimulates natural killer cell activity and interferon gamma production in T-helper type I cells. IL-18 binding protein (IL18BP) is a constitutively expressed and secreted endogenous antagonist of IL-18. It is produced by mononuclear cells and can be upregulated by IFN-gamma, presumably as part of a feedback mechanism to downregulate IL-18 activity. Elevated levels of IL-18 and IL-18BP have been observed in patients with idiopathic thrombocytopenia purpura [25], suggesting a possible link to reduced platelet number and/or function, as in severe dengue and leptospirosis. IL18BP may be a determinant of immune response to viral infections including hepatitis C [36], and bacterial infections such as brucellosis [37]; however, no prior studies have examined IL-18BP in the context of dengue and leptospirosis. In our cohort, nearly all dengue patients and approximately half of leptospirosis patients had elevated levels of IL-18BP relative to healthy norms and IL-18BP was further elevated in dengue compared to leptospirosis. This finding may be explained by the increased levels of IFN-gamma observed in both dengue and leptospirosis [22,38], which may serve to stimulate IL-18BP production. IL-18BP may play an immunomodulatory role in these inflammatory conditions, although further studies are needed to elucidate a causal role for IL-18BP in limiting disease progression. Quantitative IL-18BP levels were higher in dengue than leptospirosis and could accurately be used to differentiate the two infections, both as an individual quantitative biomarker and in combination with other clinical and biochemical features.

In addition to identifying serum proteins that distinguish between dengue and leptospirosis, our findings provide noteworthy insights into numerous host pathways that are engaged during infection with dengue and leptospirosis. We examined several categories of host response to infection, including endothelial activation or quiescence, inflammation, coagulation, and the complement system. These factors have previously been studied in the context of malaria [16,17,20], sepsis [18,39], hemolytic-uremic syndrome [40], HIV/AIDS [19,41]. We studied regulatory proteins involved in these pathways, as well as endothelial cell surface receptors that are abnormally shed into the circulation during endothelial activation (sTie-1, sTie-2, sFlt-1, sKDR, sEng, and sICAM-1).

Dysregulation of the vascular endothelium with plasma leakage plays a defining role in dengue shock syndrome [42]. Leptospirosis is also associated with vascular injury and endothelial pathology that may contribute to the frank hemorrhages that characterize Weil syndrome (severe leptospirosis) [43]. The angiopoietins (Angs) and their tyrosine kinase receptors (Tie) are regulators endothelial activation or quiescence in mature vascular beds [44,45], and vascular permeability. Previous investigators have found derangements in Ang-1 and Ang-2 in patients with severe dengue infection that are associated with plasma leakage [46]. In our study, Ang-1 levels were lower in dengue patients than healthy controls (p = 0.017) but were not different between individuals with leptospirosis and healthy controls (p = 0.107). Low levels of Ang-1 may therefore contribute to endothelial activation and vascular leak in dengue infection.

Angiopoeitin-like-3 (Angptl3) and Angiopoietin-like-4 (Angptl4) are secreted glycoproteins which share sequence homology with the angiopoietins. Unlike the angiopoietins, Angptls do not bind Tie-1 or Tie-2 and their cognate receptors are unknown. Circulating levels of Angptl3 and Angptl4 have not previously been studied in dengue or leptospirosis. In our study, we found elevated levels of Angptl3 in patients with dengue fever relative to healthy controls and leptospirosis, whereas Angptl4 levels were similar in the two patient groups and did not differ from healthy controls. Angptl3 plays a role in lipid metabolism [47], hematopoietic stem cell activity [48], angiogenesis [49], and endothelial permeability in the glomerulus [50]. Our findings suggest that Angptl3 may participate in pathologic processes in dengue virus infection, with a possible role in modulating endothelial permeability, a hallmark of severe dengue.

We examined circulating levels of the angiopoietin tyrosine kinase receptors Tie-1 and Tie-2 and found statistically significant, albeit quantitatively modest differences in sTie-2 between dengue and leptosirosis patients, whereas sTie-1 levels were similar. Elevated levels of sTie-2 have been detected in the peripheral circulation of patients with sepsis [51], severe malaria [20,52], malignancy [53,54], and inflammatory bowel disease [55]. Soluble Tie-2 is released from the endothelial cell surface by proteolytic cleavage of the extracellular domain of the Tie-2 receptor by matrix metalloproteases [51]. In our study, levels of sTie-2 were elevated in leptospirosis relative to dengue. However, levels of sTie-2 were generally within the range of healthy controls in both conditions, suggesting that leptospirosis infection produces subtle derangements in sTie-2 levels.

Another critical regulatory pathway of endothelial activation is vascular endothelial growth factor (VEGF) and its tyrosine kinase receptors, Flt-1 (VEGFR-1) and KDR (VEGFR-2). KDR expressed on the endothelial cell surface, mediates most of the endothelial growth and survival signals, whereas Flt-1 acts as a negative regulator. We found that sKDR levels were higher in dengue fever than in leptospirosis. This suggests that sKDR may be useful as a biomarker to discriminate between leptospirosis and dengue.

Endothelial adhesion molecules, including ICAM-1, appear to be involved in host response to both leptospirosis and dengue. Recombinant leptospira antigens increase ICAM-1 expression on human umbilical vein endothelial cells *in vitro*[56,57]. ICAM-1 is shed from endothelial cells after exposure to the pro-inflammatory cytokines TNF and IL-1. The soluble form of ICAM-1 (sICAM-1) is increased in sepsis, severe malaria, during the febrile stage of dengue infection [34,58], and in leptospirosis [59]. In our study, sICAM-1 was elevated relative to healthy controls in both dengue and leptospirosis, as in previous studies. Our study extends these observations, demonstrating that quantitative levels of sICAM-1 are more markedly elevated in dengue relative to leptospirosis, suggesting that sICAM-1 may have diagnostic utility in differentiating the two infections.

Abnormal hemostasis is a defining feature of dengue hemorrhagic fever and Weil’s syndrome (severe leptospirosis that can be associated withpulmonary hemorrhage). Alterations in platelet number and function contribute to the coagulopathy in both diseases. Dengue infection is associated with thrombocytopenia, a positive tourniquet test [9,13], and release of platelet contents, including platelet factor 4 (PF4), into the circulation [60]. Leptospira proteins bind fibrinogen and block platelet aggregation [61]. In our study, the platelet count was reduced in both dengue and leptospirosis, with more profound thrombocytopenia in dengue fever. A positive tourniquet test was found in 75% of laboratory-confirmed dengue cases, versus 23% of leptospirosis cases, as has been described in other studies comparing the clinical features of dengue and leptospirosis [9,13]. Finally, the platelet alpha granule factor PF4 was increased in dengue fever relative to healthy controls, but not in leptospirosis. This suggests that levated levels of PF4 (>40,000 ng/mL) may be useful for positively identifying patients with dengue infection. Of note, another phylogenetically ancient role for PF4 is as a CXC chemokine that participates in innate host defenses, forming immunogenic neoantigens by binding polyanions including components of foreign pathogens [24]. Our finding of elevated levels of PF4 in dengue, but not leptospirosis, suggests a role of PF4 as a mediator of innate immunity or pathogenesis in dengue virus infection. Further studies are warranted to test this hypothesis.

The complement system appears to play an important role in innate immune control of both dengue virus and leptospires. Dysregulation of the alternative complement pathway is associated with severe dengue infection [62]. Furthermore, the mannose binding lectin (MBL) pathway of the complement system has been implicated in controlling dengue virus infections and modulating disease manifestations [63]. The alternative pathway of the complement system also plays a central role in innate defense against leptospirosis, as illustrated by the sensitivity of non-pathogenic and resistance of pathogenic *Leptospira* species to the cidal activity of human serum [64,65]. We evaluated components of the complement system, including the anaphylatoxin C5a and Factor D, a trypsin peptidase involved in the alternative pathway of complement system activation. Factor D was significantly elevated in patients with dengue, relative to leptospirosis and relative to healthy controls. Factor D has previous been shown to be elevated in patients with DHF compared to uncomplicated dengue fever, suggesting an important role for Factor D in the immunopathology of DHF, perhaps through the amplification of downstream complement factors and inflammatory mediators. Our study lends support to these findings and additionally suggests that elevated Factor D levels in dengue infection may serve to distinguish it from leptospirosis.

Our investigation into diagnostic biomarkers for dengue and leptospirosis is subject to several limitations. Because patients were sampled from a single site (Bucaramanga, Columbia), and biomarker levels may vary according to genetic background, age, presence of co-infections, nutritional status, pre-existing immunity, or other environmental factors, results should be extrapolated with caution to different geographic areas or different demographic groups. Whereas our study used well-defined patient samples with dengue or leptospirosis, other pathogens may confound the clinical diagnosis in different settings (e.g., malaria or scrub typhus). It would be important to validate our findings in different areas and patient populations, and across a range of pathogens with a similar clinical presentation to examine the robustness as well as the specificity and predictive value of diagnostic host biomarkers. Ideally, biomarker levels should also be tested across the full spectrum of disease manifestations, including severe dengue and Weil syndrome. Finally, a larger sample of healthy control patients would yield more precise estimates of the upper and lower normal limits of the serum level of these novel proteins.

Despite similarities in their clinical presentation, dengue (an intracellular virus) and leptospirosis (an extracellular bacterium) differ microbiologically, such that divergent host responses to these pathogens might be exploited to develop tools to discriminate between the infections in clinical practice. Our work examined a broad and diverse panel of host proteins, demonstrating that sEng and IL18BP in particular were differentially upregulated in dengue relative to leptospirosis and healthy controls. Detection of elevated levels of these biomarkers strongly points to dengue virus at the likely etiology in patients with the typical, overlapping clinical presentation.

## Conclusion

Following validation in independent patient populations, these data may accelerate the development of simple clinical instruments, such as point-of-care lateral flow immunochromatographic rapid tests that could be widely implemented in resource-limited settings where dengue and leptospirosis co-circulate. Our findings also produced noteworthy insights into the activation of diverse host response programs, generating novel hypotheses into the pathogenesis of dengue and leptospirosis.

## Abbreviations

Ang-1: Angiopoietin-1; Ang-2: Angiopoietin-2; sTie-2: Soluble tie-2; VEGF: Vascular endothelial growth factor; sVEGFR-1/sFlt-1: Soluble VEGF receptor 1; sVEGFR-2/sKDR: Soluble VEGF receptor 2; sEng: Soluble endoglin; CRP: C-reactive protein; IL-10: Interleukin-10; IL-18BP: Interleukin-18 binding protein; IP-10/CXCL10: 10 kDa-interferon induced protein; sICAM-1: Soluble intercellular adhesion molecule 1; CHI3L1: Chitinase 3-like 1; C5a: Complement factor 5a; Factor D: Complement factor D; Angptl3: Angiopoietein-like 3; Angptl4: Angiopoietin-like 4; PF4: Platelet factor 4; ROC: Receiver operating characteristic; AUC: Area under the curve; CI: Confidence interval.

## Competing interests

The authors declare that they have no competing interests.

## Authors’ contributions

ALC, MG, MH, NR, WCL, LAVC and KCK participated in the design of the study. ALC, MG and NR carried out the immunoassays. ALC and MH performed the statistical analysis. WCL, LAVC and KCK contributed reagents/materials/analysis tools. ALC, MH and KCK wrote the initial draft of the manuscript. MG, NR, WCL and LAVC critically revised the manuscript. All authors read and approved the final manuscript.

## Pre-publication history

The pre-publication history for this paper can be accessed here:

http://www.biomedcentral.com/1471-2334/14/35/prepub

## Supplementary Material

Additional file 1 **Table S1 and Table S2 accompany the manuscript.** They provide statistical validation for the two logistic regression models presented in Figure 1. Click here for file
