# Supplementary material for: Host biomarkers distinguish dengue from leptospirosis in Colombia: a case–control study
Source: BMC Infect Dis. 2014 Jan 20;14:35. doi: 10.1186/1471-2334-14-35 (PMC3909480; doi:10.1186/1471-2334-14-35)
Supplement: Additional file 1 — Table S1 and Table S2 accompany the manuscript. They provide statistical validation for the two logistic regression models presented in Figure 1. [file 1471-2334-14-35-S1.docx]

**Supplementary Table 1: Validation of Logistic Regression Model for Clinical and Laboratory Parameters**

| Variables entered into model | B | S.E. | Wald | df | Sig. | Exp(B) | 95.0% C.I. for EXP(B) | |
| --- | --- | --- | --- | --- | --- | --- | --- | --- |
|  |  |  |  |  |  |  | Lower | Upper |
| Age | -.024 | .018 | 1.802 | 1 | .179 | .977 | .944 | 1.011 |
| Sex | .063 | .549 | .013 | 1 | .909 | 1.065 | .363 | 3.121 |
| Height | .029 | .019 | 2.191 | 1 | .139 | 1.029 | .991 | 1.069 |
| Duration of fever | -.020 | .013 | 2.425 | 1 | .119 | .980 | .956 | 1.005 |
| Leukopenia | 2.592 | .580 | 19.946 | 1 | .000 | 13.360 | 4.283 | 41.674 |
| Rash | 2.170 | .658 | 10.884 | 1 | .001 | 8.760 | 2.413 | 31.802 |
| Dizziness | -1.959 | .666 | 8.639 | 1 | .003 | .141 | .038 | .521 |

Model specifications: -2 Log likelihood, 115.35; Cox & Snell R Square, 0.314; Nagelkerke R Square 0.451; Hosmer and Lemeshow Test (Chi-square, 7.407; df, 8; p=0.493)

C-index: 0.857

**Supplementary Table 2: Validation of Logistic Regression Model for Clinical and Laboratory Parameters Including Host Biomarkers**

| Variables entered into model | B | S.E. | Wald | df | Sig. | Exp(B) | 95.0% C.I. for EXP(B) | |
| --- | --- | --- | --- | --- | --- | --- | --- | --- |
|  |  |  |  |  |  |  | Lower | Upper |
| Age | -.002 | .031 | .005 | 1 | .944 | .998 | .938 | 1.061 |
| Sex | -1.619 | 1.191 | 1.849 | 1 | .174 | .198 | .019 | 2.044 |
| Height | .025 | .038 | .433 | 1 | .510 | 1.025 | .952 | 1.105 |
| Duration of fever | .000 | .025 | .000 | 1 | .997 | 1.000 | .952 | 1.050 |
| High IL18BP | 3.533 | .945 | 13.968 | 1 | .000 | 34.211 | 5.366 | 218.130 |
| High sEng | 4.018 | 1.074 | 13.993 | 1 | .000 | 55.574 | 6.771 | 456.151 |
| Leukopenia | .938 | .988 | .901 | 1 | .342 | 2.555 | .369 | 17.708 |
| Rash | 2.192 | 1.039 | 4.454 | 1 | .035 | 8.950 | 1.169 | 68.519 |
| Dizziness | -2.326 | 1.162 | 4.007 | 1 | .045 | .098 | .010 | .953 |

Model specifications: -2 Log likelihood, 46.19; Cox & Snell R Square, 0.578; Nagelkerke R Square 0.832; Hosmer and Lemeshow Test (Chi-square, 4.719; df, 8; p=0.787)

C-index: 0.979
